# SEC23A Is an Independent Prognostic Biomarker in Bladder Cancer Correlated With MAPK Signaling

**DOI:** 10.3389/fgene.2021.672832

**Published:** 2021-08-11

**Authors:** Bin Zeng, Qiting Zhao, Zhiwei Sun, Doudou Liu, Hao Chen, Xiaoshuang Li, Jianyu Wang, H. Rosie Xing

**Affiliations:** ^1^Institute of Life Sciences, Chongqing Medical University, Chongqing, China; ^2^State Key Laboratory of Ultrasound Engineering in Medicine Co-Founded By Chongqing and the Ministry of Science and Technology, School of Biomedical Engineering, Chongqing Medical University, Chongqing, China

**Keywords:** SEC23A, bladder cancer, biomarker, MAPK, MEF2A

## Abstract

Clinical data mining and bioinformatics analysis can be employed effectively to elucidate the function and underlying mechanisms of the gene of interest. Here, we have proposed a framework for the identification and validation of independent biomarkers in human cancer and for mechanistic profiling using gene sets enrichment analysis and pathway analysis. This is followed by validation with *in vitro* experiments. Using this framework to analyze the clinical relevance of SEC23A, we have discovered the prognostic potential of SEC23A in different cancers and identified SEC23A as an independent prognostic factor for poor prognosis in bladder cancer, which implicates SEC23A, for the first time, as an oncogene. Bioinformatic analyses have elucidated an association between SEC23A expression and the upregulation of the MAPK signaling pathway. Using the T24 human bladder cell line, we confirmed that knockdown of SEC23A expression could effectively impact the MAPK signaling pathway. Further, through PCR verification, we showed that MEF2A, one of the key genes of the MAPK signaling pathway, might be a downstream factor of the SEC23A gene.

## Introduction

Tumor metastasis is responsible for the majority of cancer-associated mortalities (Gupta and Massagué, 2006). Despite the improvements in cancer diagnosis and treatments, the lack of effective predictive molecular markers for monitoring disease progression and prediction of the outcome of treatment has resulted in unsatisfactory clinical outcomes for most cancers. Therefore, the identification of new independent molecular markers that can provide a more accurate prognosis is key to the outcome of individualized treatment therapy for tumors.

SEC23A is involved in the components of the coat protein complex II, which mediates the transport of most secretory proteins from the endoplasmic reticulum to the Golgi apparatus (Jing et al., 2019). Previous studies have revealed involvement of SEC23A in Cranio-lenticulo-sutural dysplasia, an autosomal recessive disease with defects in collagen secretion, which leads to craniofacial and skeletal malformations (Boyadjiev et al., 2006, 2011). Few published studies by others have shown an inhibitory role of SEC23A in human cancer progression thus its expression is correlated with favorable clinical outcomes including prostate cancer (Szczyrba et al., 2011), colorectal cancer (Li et al., 2016), melanoma (Sun et al., 2018, 2020), and breast cancer (Korpal et al., 2011). However, the mechanism underlying SEC23A regulation of human cancer progression remains largely unknown.

Clinical data mining and bioinformatics analysis is an economical approach that can be effectively employed to derive the function and underline mechanisms of the gene of interest prior to labor-intensive and costly biological experimentations. Data mining of the Cancer Genome Atlas (TCGA) offers a possibility to study the commonalities and differences of diverse cancers. Analysis of the TCGA datasets may identify new clinically relevant diagnostic or prognostic markers for mechanistic validation. Since the new mechanistic insights are derived from clinical data and bear diagnostic or/and prognostic significance, once validated biologically, the identified markers can go directly for clinical validation for its usage in a specific cancer type, or broadly.

Here, we proposed a framework for the identification and validation of independent biomarkers in human cancer via prognostic analysis of TCGA database using the R software followed by validation with *in vitro* experiments. Using SEC23A, a gene with limited mechanistic understanding in human cancer as an example, we illustrated the validity and effectiveness of this approach to derive new mechanistic understandings underlying its prognostic significance.

We report that SEC23A mRNA expression is associated with the overall survival (OS), disease-specific survival (DSS), and progression-free interval (PFI) in some cancers such as adrenocortical carcinoma, bladder urothelial carcinoma (BLCA), cervical cancer, kidney renal papillary cell carcinoma, and uveal melanoma. More importantly, multivariate analysis discovers that SEC23A expression is an independent prognostic factor for poor prognosis in BLCA. This finding implicates SEC23A, for the first time, in that that it can also function as an oncogene. By conducting multi-dimensional analysis, we ruled out remodeling of the tumor microenvironment (TME) by the SEC23A-regulated secretome as the mechanism that underlies the oncogenic activity of SEC23A. Rather, through gene set enrichment analysis (GSEA) and pathway analysis, we have identified an association between SEC23A expression and the upregulation of the MAPK signaling pathway, the best characterized intracellular mediator of oncogenes. Using the T24 human bladder cell line, we confirmed that knockdown of SEC23A expression effectively inhibits MAPK signaling. Further, we identified MEF2A, one of the key genes of the MAPK signaling pathway, might be a downstream factor of the SEC23A gene.

## Materials and Methods

### Description of Data Resources

In total, 33 clinical cancers datasets, containing tumor samples and comparable normal tissue samples from cancer patients, were downloaded from the publicly available TCGA. Each dataset includes raw counts of RNAseq expression data and corresponding clinical information. To prepare the datasets for bioinformatics analysis, insufficient or absent data on age, TNM stage, and the OS time were excluded from our analyses. Thereafter, RNA sequencing data were converted to the expression matrix numbered by a gene symbol. Next, tumor tissues were grouped according to SEC23A mRNA expression for further multi-dimentional analysis.

Tumor mutational burden (TMB), microsatellite instability (MSI), and stemness scores were downloaded from the publicly available UCSC Xena^1^. Drug sensitivity was downloaded from CellMiner^2^.

### Survival and Expression Analysis

SEC23A mRNA expression was extracted through the Perl package and was divided into different groups according to the level of SEC23A expression. Differential expressions of SEC23A in different tumor (T), node stage (N), and metastasis (M) stages were analyzed using the *T*-test. Univariate analysis of differential SEC23A expression in Pan-cancer was carried out using the R package survival. Pearson correlation coefficient analysis was conducted to determine the correlation between SEC23A expression and other data. Evaluation of the effect of SEC23A expression level and other clinical data on the survival was analyzed by using multivariate cox with the R package survival. Kaplan–Meier (KM) analysis was employed to derive the OS, DSS, and PFI curves according to the median expression level of SEC23A with the R package survival.

### Using CIBERSORT and ESTIMATE to Analyze Tumor Microenvironment

We employed CIBERSORT and ESTIMATE to analyze the correlation between SEC23A mRNA expression levels and changes in the TME. CIBERSORT^3^, providing the computing source code in R, is an analytical tool based on a deconvolution algorithm that can accurately and quickly analyze gene expression profile data of complex tissues. We applied the CIBERSORT tool and the R language programs to analyze the relationship between clinical outcomes and the lymphocyte infiltration ratio of 22 distinct cell types, several of which have been associated with tumor growth, tumor progression, and outcome (Mantovani et al., 2004; Hanahan and Weinberg, 2011; Coussens et al., 2013; Gentles et al., 2015; Newman et al., 2015). ESTIMATE can predict tumor purity and the proportion of infiltrating stromal cells and immune cells in the tumor tissue (Yoshihara et al., 2013). The ESTIMATE scores (stromal and immune scores) were downloaded from the ESTIMATE online platform, matched to gene expression and clinical data by cBioPortal using sample ID codes. We used ESTIMATE and CIBERSORT to calculate the ratio of immune matrix components for each sample, the immune scores, the abundance of immune cell sub-population, and their respective correlation with SEC23A expression by the R language programs, followed by prognostic analyses.

### Gene Set Enrichment Analysis

Gene set enrichment analysis was used to generate an initial list of gene taxa relate to the SEC23A expression (Subramanian et al., 2007). This calculation illustrated a significant difference in survival between the high SEC23A expression group and the low SEC23A expression group. The GSEA software written in JAVA was used for GSEA. The expression level of SEC23A was the phenotype label of our interest. In total, 1,000 random sample permutations were performed, and nominal *p*-value < 0.05, false discovery rate (FDR) < 25%, FDR < 0.05, and normalized enrichment score (NES) were considered as significant (Subramanian et al., 2005).

### Cell Culture and Lentivirus Production

The human bladder cancer T24 cell line was obtained from China Center for Type Culture Collection. T24 cells were cultured in MEM medium (Hyclone) with 10% fetal bovine serum (Gibco) and 1% penicillin-streptomycin (Hyclone) and maintained in 37°C with 5% CO_2_ incubator. The sequence for the sh-RNAs targeting SEC23A was 5′-GGAAGCTACAAGAATGGTTGT-3′. The lentivirus particles of shSEC23A were prepared by Sangon Biotech Co.

### RT-qPCR

TRIZOL, PrimeScript RT Master Mix and SYBR Green Real-time PCR Master Mix kit were purchased from Takara, Japan. PCR reactions were set up in a 10 μl reaction volume and performed following the manufacturer’s instructions: 39 cycles of PCR amplification were performed using 95°C for 30 s, 95°C for 5 s, and 60°C for 30 s for each cycle. TBP was used as a loading control. The primer sequences of SEC23A were AGTGGCGGAGTCAGGATAC (forward) and GGCATTGGAAATCTGGAGTG (reverse); the primer sequences of TBP were TATAATCCCAAGCGGTTTGCA (forward); and CACAGCTCCCCAC CATATTC (reverse).

### Western Blotting

A RIPA buffer with 1% PMSF (Beyotime) was used to extract the cell total protein, and the concentration was determined by BCA (Beyotime). For Western blotting analysis, protein samples were boiled for 10 min. In total, 20–40 μg of the protein was run on a 12% polyacrylamide (Beyotime) gel and transferred to a PVDF membrane (Millipore). The membrane was first blocked in QuickBlock^TM^ Western Seal Fluid (Beyotime), followed by incubation with the primary and secondary antibodies, respectively, in accordance with the manufacturer’s instructions: Primary antibodies were incubated overnight at 4°C; then, the second antibody was added and incubated at room temperature for 1 h. The following primary antibodies were used: SEC23A (CST) and anti-GAPDH (Proteintech).

### CCK-8 Assay

The cell counting kit (CCK)-8 assay was used to measure cell proliferation. Cells were plated in 96-well plates at a density of 1.5 × 10^3^ cells per well, and they were measured for six consecutive days. The absorbance of each well at 450 nm was measured with an enzyme-linked immunosorbent assay reader.

### Transwell Migration and Matrigel Invasion Assay

Both the migration and invasion assay used a Transwell chamber. The upper layer of the chamber was coated with Matrigel for invasion assay. After suspension of cells in a serum-free medium, 300 μl medium with 3–4 × 10^4^ tumor cells were added to the upper chamber, and 650 μl 10% serum-containing medium was added to the lower chamber. After overnight culture, the cells which migrated and invaded the subsurface of the membrane were fixed with cold methanol and stained with crystal violet.

### Ethical Approval

The study does not involve animals or humans. Thus, no ethical approval is required.

### Statistical Analysis

The 33 cancers patients datasets from TCGA were merged and conducted by the Perl package and the R package. Differences in gene expression between the individual groups were performed by Student’s independent *t*-test using the R package and GraphPad Prism 8 software. KM analyses, univariate analyses, and multivariate cox analyses were analyzed by the R package survival. Pearson correlation coefficient analysis was used to analyze the correlation between SEC23A expression and other data (TMB, MSI, stemness score, and drug sensitivity). Differences were considered statistically significant when *p* < 0.001 (^∗∗∗^); *p* < 0.01 (^∗∗^); and *p* < 0.05 (^∗^).

## Results

### The Prognostic Value of SEC23A Expression

**FIGURE 1:**
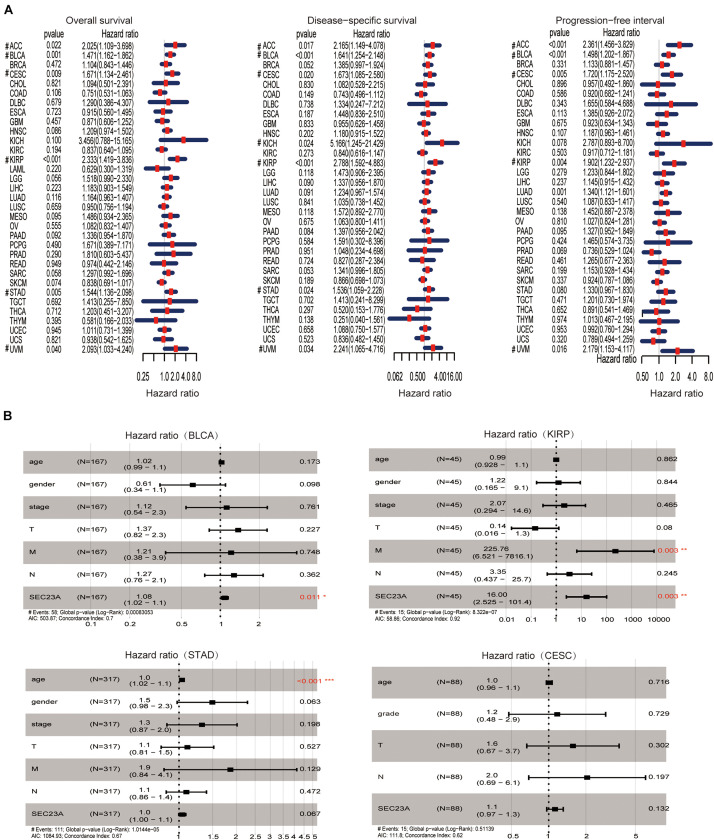
Prognostic value of SEC23A in pan-cancer patients and Multivariate Cox analysis in BLCA, KIRP, STAD, and CESC. **(A)** Univariate analysis of SEC23A in Pan-cancer patients (OS, DSS, and PFI; *p* < 0.05). **(B)** Multivariate Cox analysis of SEC23A expression and other clinical-pathological data (*p < 0.01; **p < 0.01; and ***p < 0.001).

Using hash and survival packages in the R program, we performed univariate cox regression for survival analysis in different cancer types. We observed that the high expression of SEC23A is positively associated with poor outcomes in OS, DSS, and PFI in BLCA, ACC, CESC, KIRP, and UVM patients (Figure 1A; Supplementary Table 1). The four cancers with *p* < 0.01 (OS) in the univariate analysis were selected for multivariate analysis. From multivariate Cox analysis of the correlation between the level of SEC23A expression and other clinical or pathological parameters, we identified for the first time that SEC23A was an independent poor prognostic factor in BLCA and KIRP (Figure 1B). This finding suggests that unlike the tumor suppressor function of SEC23A, all literature reported thus far, SEC23A may also act as an oncogene in certain cancer types. However, after eliminating samples with missing data in KIRP, the number of patients with this condition (45 cases) was too small to confirm the prognostic value. Thus, in the present study, we focused our validation on the oncogenic role of SEC23A in bladder cancer.

### SEC23A Expression Comparison Analysis in Bladder Cancer

**FIGURE 2:**
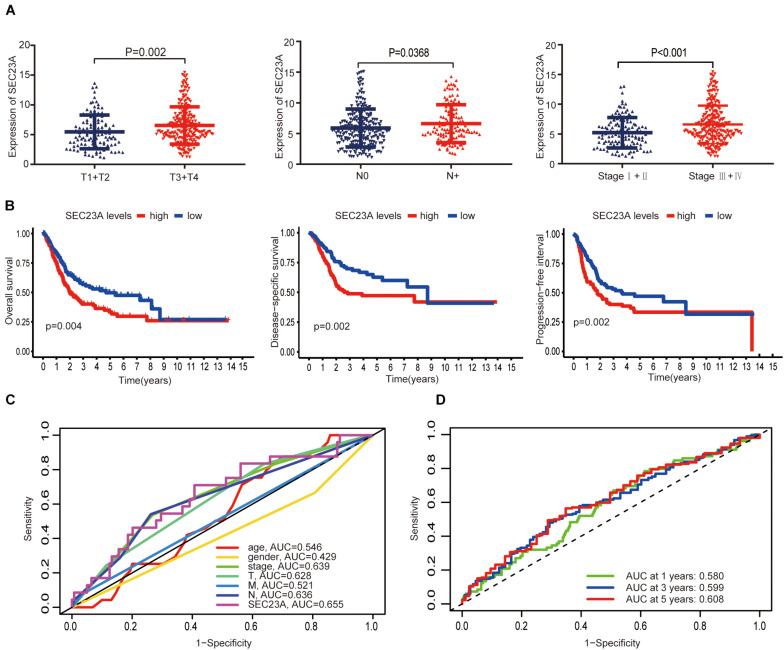
Differential expression of SEC23A in bladder cancer and ROC analysis of SEC23A expression. **(A)** Association between SEC23A and clinical-pathological features. **(B)** Survival analysis of SEC23A (OS, DSS, PFI, and K-M analysis). **(C)** Multi-indicator ROC analysis based on SEC23A, with TNM stages, gender, and age. **(D)** Time-dependent ROC analysis on TCGA cohorts based on SEC23A.

In December 2020, the TCGA database contains 433 clinical and gene expression data of bladder cancer, including 414 tumor samples and 19 normal samples. The median follow-up time was 15.8 months, ranging from 0 to 169 months. As shown in Figure 2, *SEC23A* expression in advanced bladder tumor samples was significantly higher than that in early stage tumor samples (T1+2 vs T3+4, N0 vs N+, Stage I + II vs Stage III + IV; *p* < 0.05; Figure 2A). Next, we performed KM analysis of SEC23A expression level and survival in bladder cancer patients. Higher SEC23A expression was significantly associated with poor OS, DSS, and PFI, which were consistent with univariate analysis (Figure 2B). Multi-indicator ROC analysis showed that SEC23A gave the highest AUC value (AUC = 0.636; Figure 2C). Time-dependent ROC analysis showed that the 5-year AUC of the score model was 0.608, which is relatively higher than that of the 1 year (AUC of 0.580) and the 3 years (AUC of 0.599; Figure 2D).

### Association of SEC23A Expression With TMB, MSI, Stemness Score, Drug-Sensitivity, and Tumor Microenvironment

**FIGURE 3:**
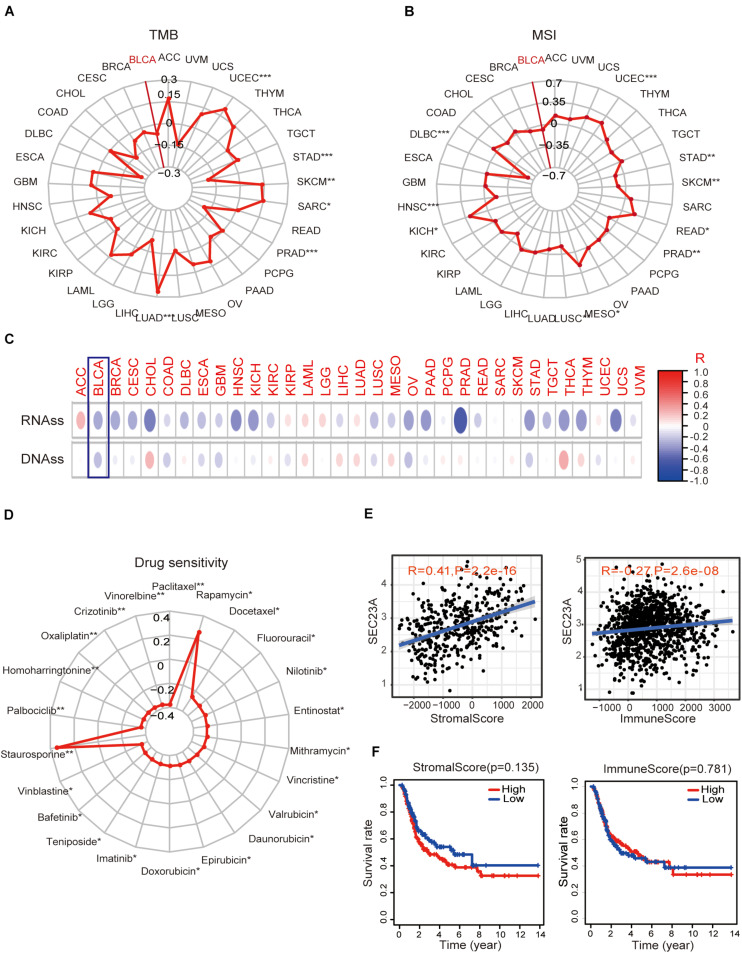
Association of SEC23A expression with TMB, MSI, stemness score, drug sensitivity, and TME. **(A,B)** No significant correlations between SEC23A expression level and TMB and MSI in Pan-cancer patients, even though the *p*-value was statistically significant (p*** < 0.001; p** < 0.01; and p* < 0.05). **(C,D)** No significant correlations between SEC23A expression level and cancer stem cells and drug sensitivity (Antitumor agents) even though the *p*-value was statistically significant (p*** < 0.001; p** < 0.01; and p* < 0.05). **(E)** SEC23A expression level significantly correlated with Stromalscore and Immunescore. **(F)** Survival analysis of SEC23A associated with levels of Stromalscore and Immunescore. Pearson correlation was applied to analyze the correlation.

Next, we performed bioinformatic virtual analysis to objectively derive mechanisms underlying the oncogenic activity of SEC23A in human bladder cancer (section “Materials and Methods”). The correlations between SEC23A expression level and TMB, MSI, stemness score, and drug sensitivity (Antitumor agents) were too low to be considered significant in all cancer types included in the analysis (section “Materials and Methods,” Figures 3A–D; Supplementary Table 2).

Since the well-characterized function of SEC23A is its regulation of secretory proteins (Jing et al., 2019), we investigated whether SEC23A expression is associated with TME remodeling by examining the matrix deposit (the Stromalscore) and tumor-infiltrating immune cells (TIIC, the Immunescore) in BLCA. We observed that SEC23A expression levels significantly correlated with Stromalscore and Immunescore via the ESTIMATE (Yoshihara et al., 2013) packages in R program analysis (Figure 3E) and found no significant prognosis (Figure 3F). Further, we analyzed the association between SEC23A expression and the changes in TIIC concentration in a bladder cancer sample via the CIBERSORT (Newman et al., 2015) and observed a weak association that was not statistically significant (Supplementary Figure 1). Collectively these findings ruled out TMI remodeling by SEC23A-regulated secretome as the mechanism of the oncogenic activity of SEC23A.

### Gene Sets Enriched in SEC23A Expression Phenotype

**FIGURE 4:**
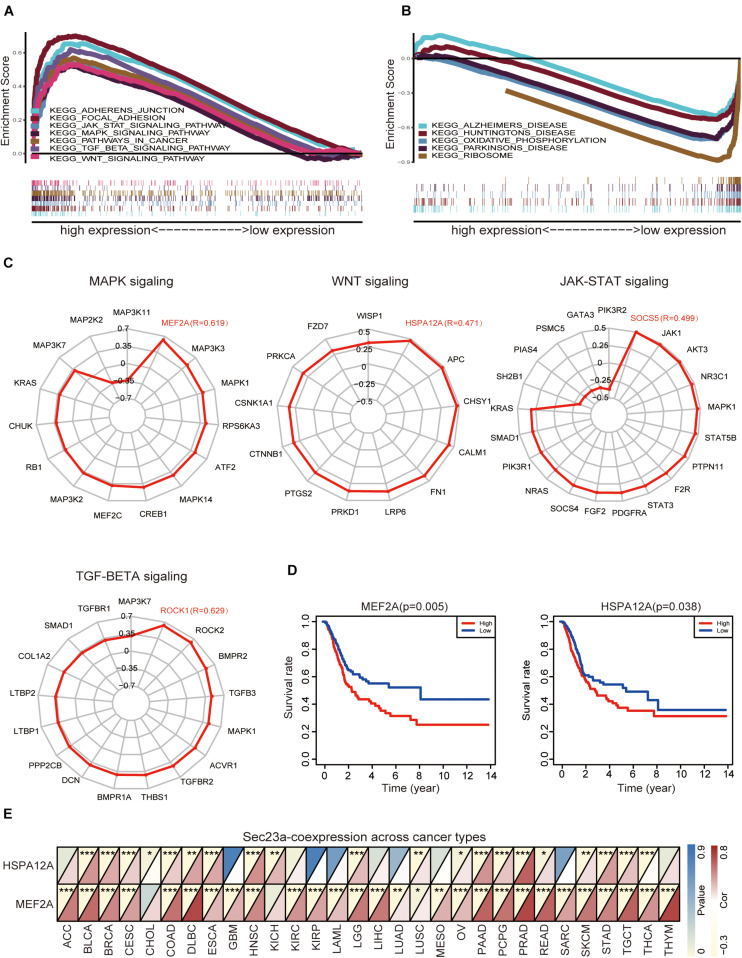
GSEA analysis prioritized differentially enriched genes in KEGG that correlated with SEC23A expression. **(A)** HIGH expression in SEC23A. **(B)** LOW expression in SEC23A. **(C)** The correlation between SEC23A and Key genes of MAPK,WNT, JAK-STAT, and TGF-BETA signaling pathway (*R* ≥ | 0.3|). **(D)** KM survival analysis of MEF2A and Hspa12a base on TCGA. **(E)** Pearson analysis of MEF2A and Hspa12a related to SEC23A in Pan-cancer patients. MEF2A has a higher level of correlation (*p < 0.01; **p < 0.01; and ***p < 0.001).

We used GESA to identify SEC23A-correlated molecular pathways in bladder cancer. Due to the limited space, the top seven ranked significantly and differentially enriched pathways associated with high SEC23A expression were listed here in Figure 4A (Supplementary Table 3). The top five ranked signaling pathways significantly enriched and associated with low SEC23A expression were listed in Figure 4B (Supplementary Table 3).

Next, we focused attention on the JAK-STAT signaling pathway, MAPK signaling pathway, TGF-beta signaling pathway, and WNT signaling pathway in BLCA (Figure 4A). Based on literature mining, we generated a list of genes that play key and regulatory roles in the MAPK signaling pathway (Kyriakis and Avruch, 2001; Johnson and Lapadat, 2002; Silvers et al., 2003; Muniyappa and Das, 2008), JAK-STAT pathway (Marrero, 2005; Ferrajoli et al., 2006; Schindler et al., 2007; Bray et al., 2018), TGF-beta pathway (Dennler et al., 2002; ten Dijke and Hill, 2004), and WNT pathway (MacDonald et al., 2009; Sokol, 2011; Niehrs, 2012). To determine if there was a co-expression pattern in gene expression between SEC23A and these key genes, Pearson’s correlation analysis was performed on gene expression values. We found that MEF2A, Hspa12a, Socs5, and Rock1 were most strongly associated with the SEC23A (Figure 4C; Supplementary Table 4). However, only MEF2A and Hspa12a exhibited significant prognostic values in BLCA (Figure 4D). We next evaluated the correlation between SEC23A expression and MEF2A or Hspa12a in Pan-cancer patients and prioritized MEF2A for biological validation (Figure 4E).

### SEC23A Expression Effectively Promote the Invasive Behavior of Bladder Cancer T24 Cells *in vitro*

**FIGURE 5:**
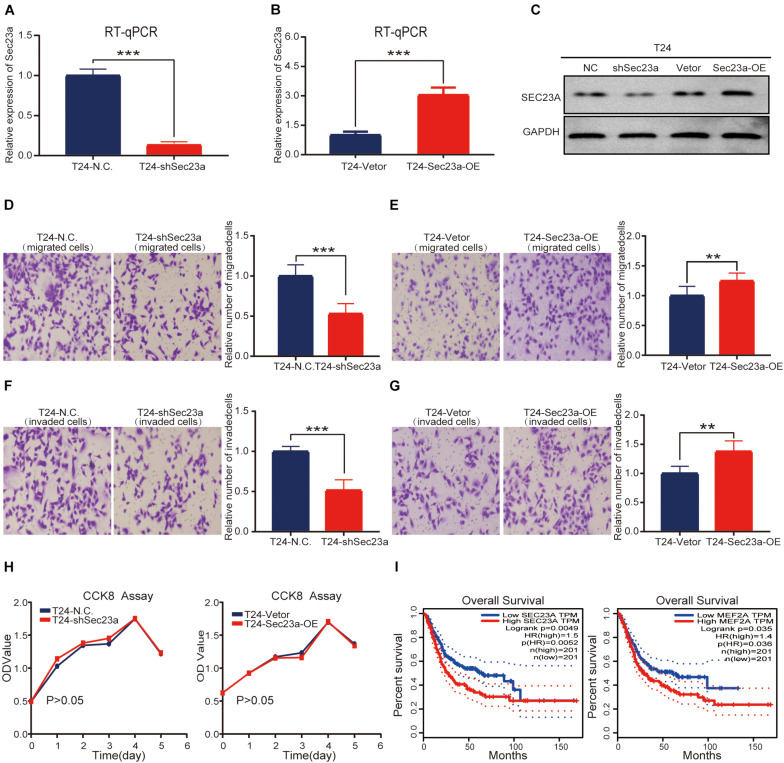
SEC23A expression effectively augments the metastatic capacities of T24 *in vitro*. **(A)** RT-PCR measurement of mRNA expression of SEC23A in T24 and T24-shSEC23A cells; **(B)** RT-PCR measurement of mRNA expression of SEC23A in T24 and T24-SEC23A-OE cells. **(C)** Western-blotting analysis of SEC23A in T24, T24-shSEC23A, T24-vetor, and T24-SEC23A-OE cells. **(D)** Transwell migration assay of T24 and T24-shSEC23A cells (***p < 0.001). **(E)** Transwell migration assay of T24 and T24-SEC23A-OE cells (**p < 0.01); **(F)** Transwell invasion assay of T24 and T24-shSEC23A cells, (***p < 0.001). **(G)** Transwell invasion assay of T24 and T24-SEC23A-OE cells, (**p < 0.01). **(H)** Proliferation activities of T24 vs T24-shSEC23A and T24 vs T24-SEC23A-OE were measured by the CCK-8 assay. **(I)** Survival analysis of SEC23A and MEF2A by GEPIA.

To confirm the oncogenic activity of SEC23A we identified through bioinformatics analyses in bladder cancer, the widely used human bladder cancer cell line T24 with SEC23A overexpression or silencing was successfully constructed. Overexpression and knock-down efficiency of SEC23A were confirmed through RT-qPCR (Figures 5A,B) and western blot (Figure 5C; Supplementary Figure 2A). SEC23A silencing significantly reduced the migration and invasion capacities of T24-shSEC23A cells (Figures 5D,F). In contrast, SEC23A overexpression augmented the migration and invasion capacities of T24-SEC23A-OE (Figures 5E,G). Changes in SEC23A gene expression had no significant effect on T24 cell proliferation, compared with T24-N.C cells (Figure 5H). In order to ensure the accuracy of data analysis, we performed KM survival analysis for SEC23A and MEF2A genes by GEPIA, which was derived from the cancer genomic atlas (TCGA)^4^ (Figure 5I).

### SEC23A Expression Is Correlated With the MAPK Signaling Pathway in T24 Cells

**FIGURE 6:**
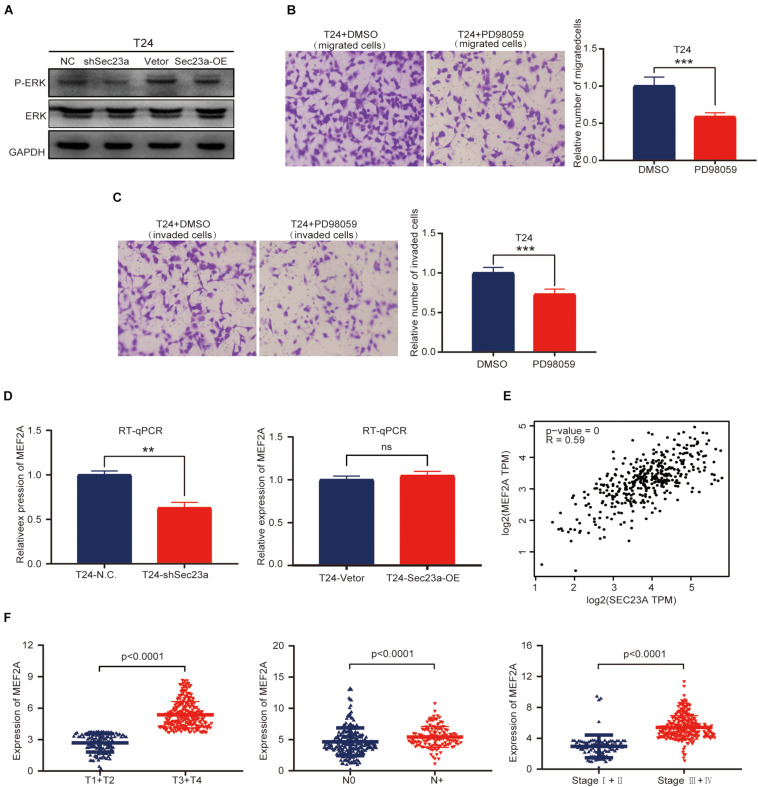
SEC23A augments ERK signaling activation in T24 cells *in vitro*. **(A)** Western-blotting analysis of p-ERK in T24 vs T24-shSEC23A, and T24 vs T24-SEC23A-OE cells. **(B)** Transwell migration assay (***p < 0.001); **(C)** Transwell invasion assay (***p < 0.001). **(D)**) RT-qPCR measurement of mRNA expression of MEF2A in T24 and T24-shSEC23A cells (**p < 0.01, **left**); and in T24 and T24-SEC23A-OE cells (**right**). **(E)** SEC23A expression level significantly correlated with MEF2A by GEPIA. **(F)** Association between MEF2A and clinical-pathological features.

Based on the above results, we focused our subsequent analysis and validation on the MAPK signaling pathway (Aksamitiene et al., 2012). The interrelationship between SEC23A and genes with prognostic value in the MAPK signaling pathway were shown in Supplementary Figure 3. To validate the predicted oncogenic activity of SEC23A on MAPK signaling, we accessed p-ERK levels in T24-shSEC23A and T24-SEC23A-OK cells, respectively. While SEC23A silencing inhibited the p-ERK (Figure 6A, left), overexpression of SEC23A did not further increase phosphorylation of ERK (Figure 6A, right; Supplementary Figure 2B). Treatment of T24 cells with PD98059, an effective and selective MEK inhibitor, significantly reduced the migration and invasion of T24 cells (Figures 6B,C). By PCR screening, we showed that the expression levels of MEF2A were significantly decreased in T24-shSEC23A (Figure 6D, left) but did not significantly change in T24-SEC23A-OE (Figure 6D, right). To ensure the accuracy of our data analysis and functional prediction, we analyzed the correlation of SEC23A with MEF2A genes by GEPIA (Figure 6E). Next, we found MEF2A gene expression in advanced tumor samples was significantly higher than that in early stage tumor samples, consistent with the oncogenic role of SEC23A in bladder cancer (Figure 6F).

## Discussion

SEC23A, a regulator of secretory protein function, plays an important role in human cancer. While few published studies reported the tumor suppressor function of SEC23A in breast, prostate, melanoma, colorectal cancer (Korpal et al., 2011; Szczyrba et al., 2011; Li et al., 2016; Sun et al., 2018, 2020), mechanistic understanding is limited.

Clinical data mining becomes increasingly important in cancer research. On the one hand, it has been used widely to identify diagnostic or prognostic-relevant preclinical findings for clinical validation and translation. On the other hand, it can predict new clinically relevant gene functions and mechanisms for biological validation. In the present study, we proposed a framework for the identification and validation of independent biomarkers in human cancer via prognostic analysis of the TCGA database using the R software, and for objective mechanistic profiling using GSEA and pathway analysis, followed by validation with *in vitro* biological experiments. As a proof of principle, we applied this framework and derived the clinical relevance of SEC23A in human cancer, especially in bladder cancer, identified SEC23A as a poor prognostic marker of bladder cancer, and predicted MAPK signaling as the mediator of the oncogenic activity of SEC23A. Most importantly, the predicted oncogenic function of SEC23A was validated with biological experimentation. Specifically, we made the following new findings:

First, this is the first study to demonstrate that SEC23A mRNA expression is associated with the OS, DSS, and PFI in different cancers such as ACC, BLCA, CESC, KIRP, and UVM patients.

Second, prior to this study, only the tumor suppressor function of SEC23A has been reported in preclinical studies when SEC23A was the target gene of miRNAs (Boyadjiev et al., 2006; Korpal et al., 2011; Szczyrba et al., 2011; Li et al., 2016; Sun et al., 2018, 2020). The clinical relevance of SEC23A was not investigated. We performed an in-depth bioinformatics analysis to evaluate the prognostic value of SEC23A in human cancers. Multivariate analysis has discovered that SEC23A expression is an independent poor prognostic factor in bladder cancer. This finding, for the first time, implicates SEC23A as an oncogene.

Third, we performed multi-dimensional bioinformatics analysis to predict the mechanisms underlying the oncogenic function of SEC23A. We first ruled out TMB, MSI, tumor stem cells, and drug sensitivity as potential mechanisms. Thereafter, we analyzed the correlation of SEC23A expression with TME remodeling by the characterized SEC23A-regulated secretom function (Heinemann et al., 2014; Nielsen et al., 2016). It is well-recognized that the TME plays a crucial role in cancer progression and treatment responses (Quail and Joyce, 2013; Annels et al., 2020). Here, we show that the expression of SEC23A was substantially related to TME, implying a potential role of SEC23A in regulating TME remodeling. However, the Stromalscore and Immunescore of TME had no correlation with the OS in BLCA patients. Therefore, the correlations of TME with SEC23A is not the mechanism that underlies the independent poor prognosis of SEC23A in bladder cancer.

Fourth, via GSEA and pathway analysis, we have identified a significant association between SEC23A expression and the upregulation of the MAPK signaling pathway (Rath et al., 2007; Degirmenci et al., 2020). Using the T24 human bladder cancer cell line, we confirmed that silencing of SEC23A expression inhibited the MAPK signaling pathway and MEF2A expression. The reverse was that overexpression of SEC23A did not further augment the level of p-ERK and MEF2A expression. This could be due to that ERK activation, measured by p-ERK, had reached an optimal state in T24 cells. We also observed that regulation of MAPK activation by SEC23A did not change T24 cell proliferation activity, while the invasive capability was affected. While MAPK activation often leads to increased cell proliferation, bladder cancer exploits the SEC23A-regulated MAPK signaling to enhance the more invasive behavior of cancer cells to confer a poor prognosis of SEC23A in bladder cancer. Thus, biological experimentation conducted in T24 cells has validated the bioinformatics predictions on SEC23A as an oncogene as an independent poor prognostic marker and uses MAPK signaling pathway to exert its oncogenic function. More extensive biological experiments are required to warrant clinical translation.

## Conclusion

Clinical data mining and bioinformatics analysis can be employed effectively to elucidate the function and underline mechanisms of SEC23A. SEC23A expression appears to be a new and independent prognostic marker for BLCA. SEC23A is correlated with the MAPK signaling pathway in BLCA. Future studies are required to elucidate the biological processes regulated by SEC23A and their respective role in cancer initiation and progression.

## Data Availability Statement

The original contributions presented in the study are included in the article/Supplementary Material; further inquiries can be directed to the corresponding author/s.

## Ethics Statement

The experiments do not involve animals or humans and do therefore not require ethical approval.

## Author Contributions

BZ and QZ performed the experiments and analyzed data, contributed to the writing of this manuscript. ZS, DL, HC, and XL participated in the conduction of this study. HX and JW designed this study, oversaw the execution of this study, and contributed to the writing and revision of this manuscript. All authors contributed to the article and approved the submitted version.

## Conflict of Interest

The authors declare that the research was conducted in the absence of any commercial or financial relationships that could be construed as a potential conflict of interest.

## Publisher’s Note

All claims expressed in this article are solely those of the authors and do not necessarily represent those of their affiliated organizations, or those of the publisher, the editors and the reviewers. Any product that may be evaluated in this article, or claim that may be made by its manufacturer, is not guaranteed or endorsed by the publisher.

## Footnotes

1
http://xena.ucsc.edu/

2
https://discover.nci.nih.gov/cellminer/home.do

3
http://cibersort.stanford.edu/

4
http://gepia.cancer-pku.cn/index.html
