# Supplementary figures and images for: SEC23A Is an Independent Prognostic Biomarker in Bladder Cancer Correlated With MAPK Signaling
Source: Front Genet. 2021 Aug 11;12:672832. doi: 10.3389/fgene.2021.672832 (PMC8385657; doi:10.3389/fgene.2021.672832)

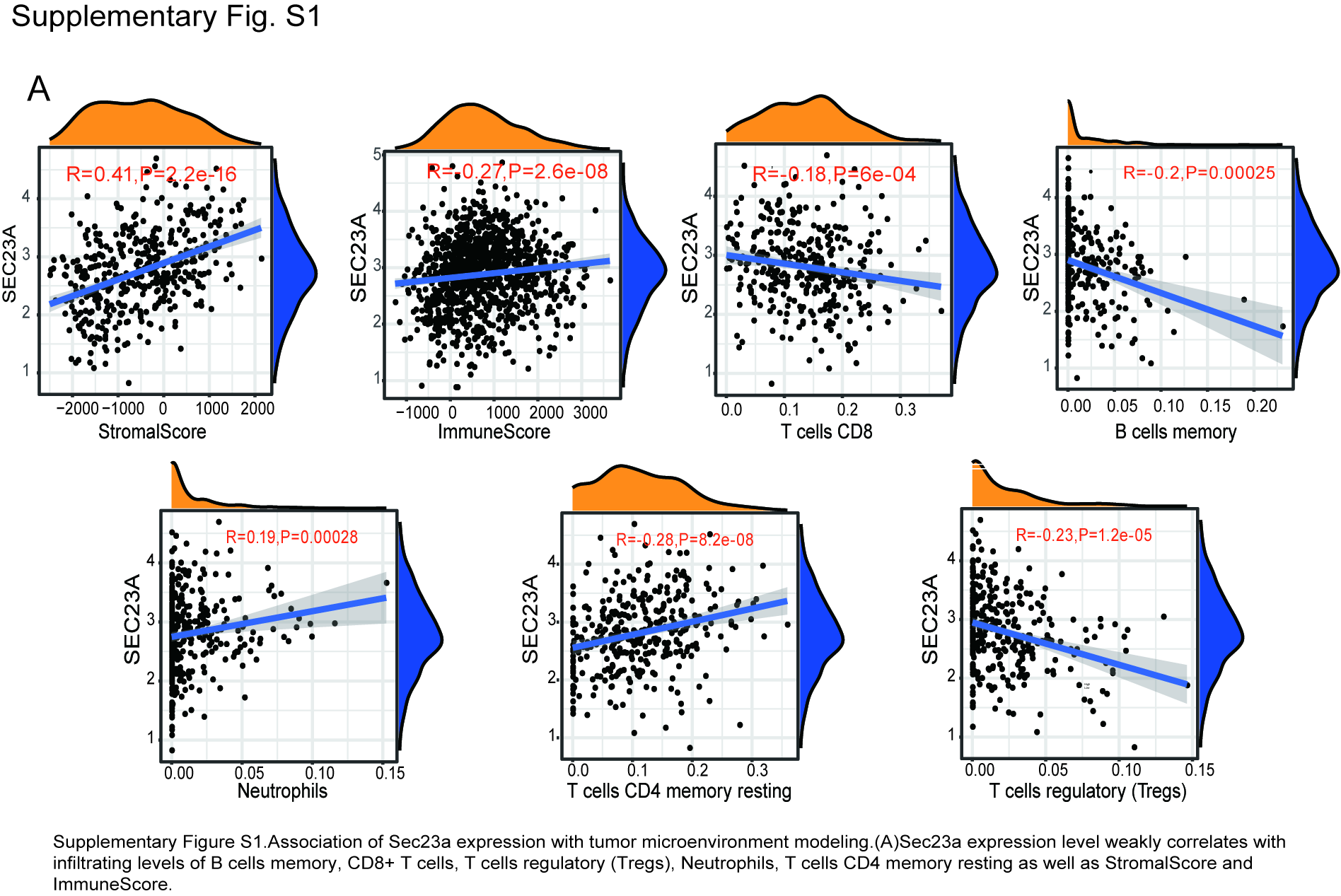

Supplement: Supplementary file 1 [file Image_1.TIF]

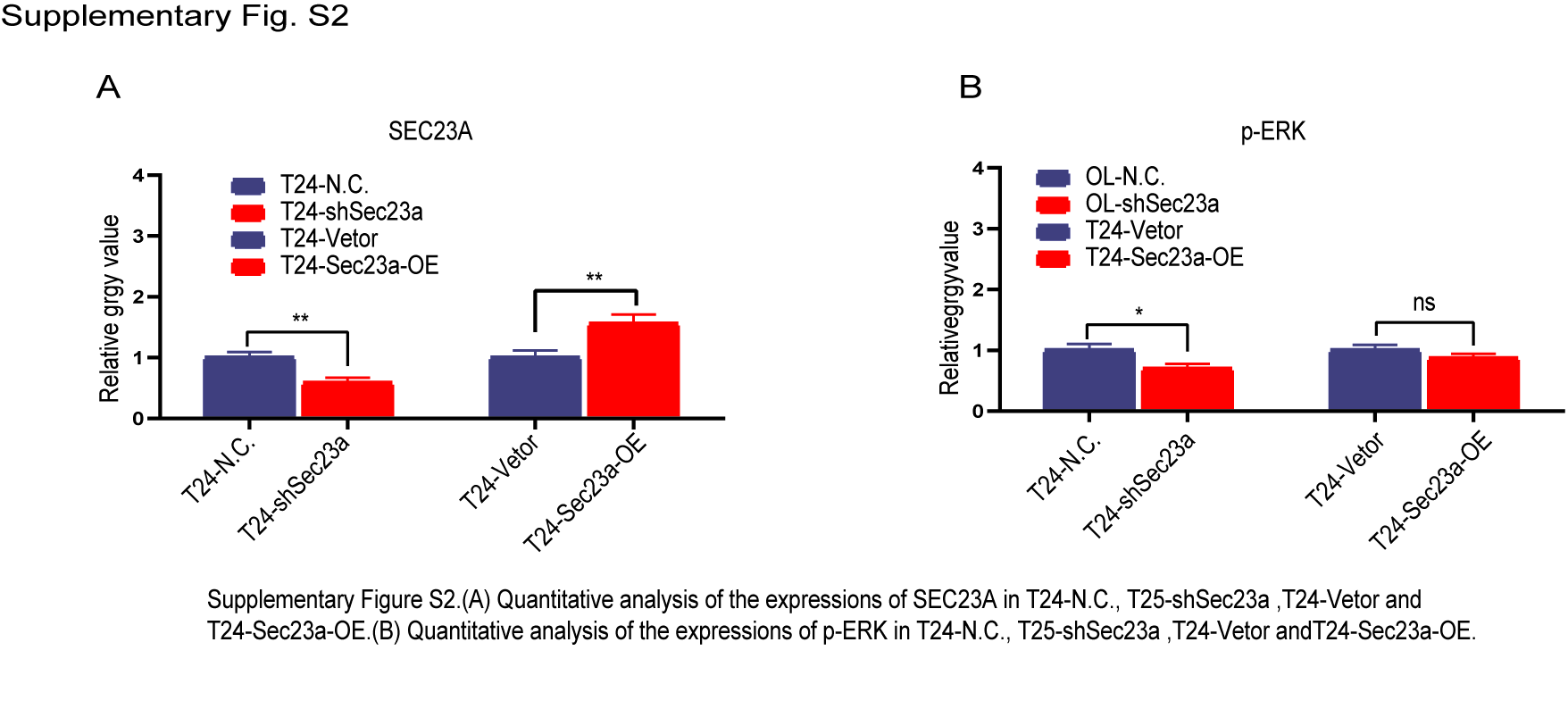

Supplement: Supplementary file 2 [file Image_2.TIF]

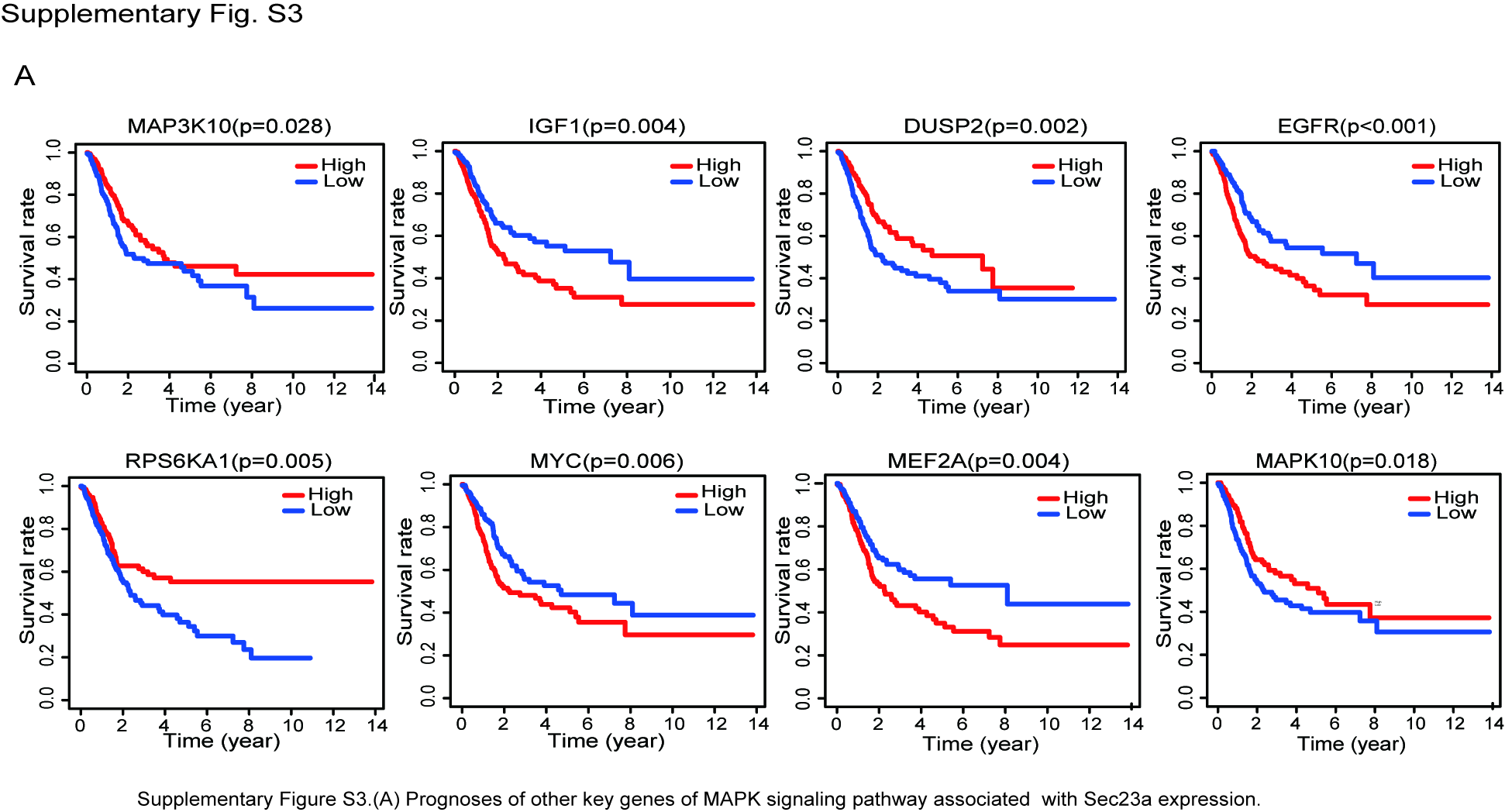

Supplement: Supplementary file 3 [file Image_3.TIF]
